# Crimean-Congo Hemorrhagic Fever Virus in Ticks from Imported Livestock, Egypt

**DOI:** 10.3201/eid1801.111071

**Published:** 2012-01

**Authors:** Katherine Chisholm, Erica Dueger, Nermeen T. Fahmy, Hamed Abdel Tawab Samaha, Alia Zayed, Mahmoud Abdel-Dayem, Jeffrey T. Villinski

**Affiliations:** US Naval Medical Research Unit No. 3, Cairo, Egypt (K. Chisholm, E. Dueger, N.T. Fahmy, A. Zayed, M. Abdel-Dayem, J.T. Villinski);; Centers for Disease Control and Prevention, Atlanta, Georgia, USA (E. Dueger);; Ministry of Agriculture, Cairo (H.A.T. Samaha)

**Keywords:** vector-borne infections, zoonoses, Crimean-Congo hemorrhagic fever, viruses, ticks, Egypt, livestock

**To the Editor:** Crimean Congo hemorrhagic fever, a tick-borne illness caused by Crimean Congo hemorrhagic fever virus (CCHFV), is endemic to Africa, the Balkans, the Middle East, and parts of Asia. The hard ticks (Ixodidae), especially those of the genus *Hyalomma*, serve as reservoirs and vectors for CCHFV, and a variety of animals, such as cattle, sheep, and camels, are considered amplifying hosts for the virus. Although CCHFV may cause little or no disease in zoonotic hosts, the virus can cause severe disease in humans who may be exposed by tick bites or by contact with blood or tissues from infected patients or animals (*1*). Surveillance for CCHFV in animal and vector populations provides an opportunity to monitor a disease of potentially severe impact.

In North Africa and the Middle East, trade in live animals, meat, and meat products poses noticeable risk to human and animal health (*2**,**3*) and can serve as a mobile pool of diseases with potentially large economic and health effects. Animals often originate in distant areas of a country or its neighbors, where they may be exposed to zoonotic pathogens not endemic to their final location, and may collect vectors that carry additional pathogens.

As part of a broader study examining occupational risk of exposure to vector-borne and zoonotic pathogens in a high-risk abattoir worker population, we collected ectoparasites from freshly-slaughtered livestock and examined them for CCHFV. This study and was conducted in compliance with the Animal Welfare Act and in accordance with the principles set forth in the Guide for the Care and Use of Laboratory Animals (*4*).

Sample collection took place over a 2-week period in July 2009 at the Muneeb abattoir in the Giza Governorate of Egypt. Ectoparasites were removed from 43 freshly slaughtered animals by using blunt forceps and were placed in glass vials. In total, 342 ectoparasites were collected: 70 (20.5%) from 14 cattle, 52 (15.2%) from 17 buffalo, 6 (1.8%) from 2 sheep, and 214 (62.6%) from 10 camels. Cattle, buffalo, and sheep originated in Egypt; camels were imported from Sudan and Somalia.

Ectoparasites were transported to US Naval Medical Research Unit No. 3 in Cairo, Egypt, for taxonomic identification and pathogen detection. Ninety-seven percent (334) of the ectoparasites were ticks from the family Ixodidae. The genus *Hyalomma* accounted for 254 (76.0%) of these ticks, including nearly all of those collected from sheep (100%) and camels (99.5%) but only 60.4% and 9.1% of those collected from buffalo and cattle, respectively. The remaining ticks of the family Ixodidae belonged to genus *Boophilus*.

After identification, ticks were grouped into pools by species, sex, and animal source. RNA was then extracted by using the QIAamp Viral RNA Kit (QIAGEN, Valencia, CA, USA). CCHFV small fragment RNA was detected with SuperScript III Platinum SYBR Green One-Step qRT-PCR (Invitrogen, Carlsbad, CA, USA) and published primers as described (*5*).

Of 138 pools tested (258 ticks of the genera *Hyalomma* and *Boophilus*), 6 pools were positive for CCHFV. These 6 pools contained ticks collected from 1 camel imported from Somalia and 4 from Sudan. One positive pool comprised female *H. excavatum* (Koch, 1844), and 5 comprised female *H. dromedarii* (Koch, 1844), both proven vectors of CCHFV.

Sequence analysis showed that each CCHFV-positive pool contained an identical yet previously unrecorded 229-bp RNA fragment (GenBank accession no. JF706233; Figure). This fragment had 92% identity with many published CCHFV isolates (BLAST analysis [www.ncbi.nlm.nih.gov/BLAST], GenBank nonredundant database); however, most base changes were synonymous substitutions. Translated protein queries (tblastx) identified an amino acid (serine at codon 26) in this new variant that differed from all but 1 (AJ010648.1) of the 100 most similar published sequences. The functional consequence of a S26N substitution is unknown but may be minimal because of the similar physicochemical properties of asparagine and serine.

**Figure:**
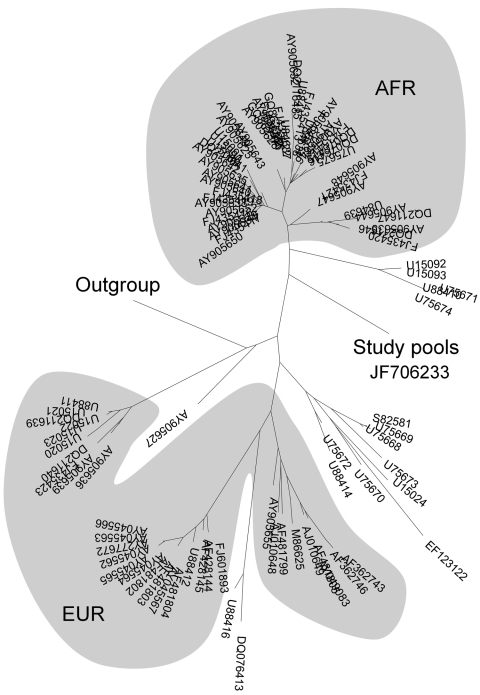
Phylogenetic relationship of 110 Crimean-Congo hemorrhagic fever virus sequences obtained from this study (6 tick pools), the positive control, and 99 published isolates from GenBank. Sequences were aligned by using ClustalX (www.clustal.org), and a phylogenetic tree was constructed by using MEGA4 (neighbor-joining, Kimura 2-parameter, complete deletion of missing data, tree condensed when bootstrap calculated branch support was <0.5) (www.megasoftware.net). The 2 shaded areas indicate clustering of African isolates (AFR) and predominantly European isolates (EUR); unshaded isolates originate primarily from the Middle East.

Despite the low number of camels sampled in this study, 5 of the 10 camels were found to harbor ticks carrying RNA from an undocumented variant of CCHF. Although none of the domestic animals harbored infected ticks, it is not possible to conclude if these data reflect importation of CCHFV or infection acquired within Egypt because details about conditions under which animals were kept before slaughter are unavailable. Previous serologic studies in Egypt have shown antibodies to CCHFV were prevalent among imported camels at a quarantine station in Aswan governorate (*6*) and among domestic cattle, buffalo, sheep, and goats in Sharkia Governorate (*7*). We plan to further investigate the presence of CCHFV within camel and ectoparasite populations in Egypt by expanding protocol activities to a camel market in the same area as the abattoir.
